# Luteinizing Hormone Action in Human Oocyte Maturation and Quality: Signaling Pathways, Regulation, and Clinical Impact

**DOI:** 10.1007/s43032-019-00137-x

**Published:** 2020-01-06

**Authors:** Armando Arroyo, Beomsu Kim, John Yeh

**Affiliations:** 1 Boston IVF - The Syracuse Center, 5792 Widewaters Pkwy., Syracuse, NY 13214 USA; 2 Department of Obstetrics and Gynecology, SUNY Upstate Medical University, 736 Irving Ave., Syracuse, NY 13210 USA; 3 CNY Fertility, 835 Hopkins Rd., Buffalo, NY 14221 USA; 4 Department of Obstetrics and Gynecology, Division of Reproductive Endocrinology and Infertility, University of Massachusetts Medical School, 119 Belmont St., Worcester, MA 01655 USA

**Keywords:** Oocyte meiotic maturation, LH follicle signaling, Oocyte quality

## Abstract

The ovarian follicle luteinizing hormone (LH) signaling molecules that regulate oocyte meiotic maturation have recently been identified. The LH signal reduces preovulatory follicle cyclic nucleotide levels which releases oocytes from the first meiotic arrest. In the ovarian follicle, the LH signal reduces cyclic nucleotide levels via the CNP/NPR2 system, the EGF/EGF receptor network, and follicle/oocyte gap junctions. In the oocyte, reduced cyclic nucleotide levels activate the maturation promoting factor (MPF). The activated MPF induces chromosome segregation and completion of the first and second meiotic divisions. The purpose of this paper is to present an overview of the current understanding of human LH signaling regulation of oocyte meiotic maturation by identifying and integrating the human studies on this topic. We found 89 human studies in the literature that identified 24 LH follicle/oocyte signaling proteins. These studies show that human oocyte meiotic maturation is regulated by the same proteins that regulate animal oocyte meiotic maturation. We also found that these LH signaling pathway molecules regulate human oocyte quality and subsequent embryo quality. Remarkably, in vitro maturation (IVM) prematuration culture (PMC) protocols that manipulate the LH signaling pathway improve human oocyte quality of cultured human oocytes. This knowledge has improved clinical human IVM efficiency which may become a routine alternative ART for some infertile patients.

## Introduction

Ovarian follicular development and its endocrine function have been the major focus of mammalian ovarian research. Ovarian follicular development has been extensively reviewed [1, 2]. The animal oocyte has received less attention and the human oocyte even less. Pincus reviewed mammalian oogenesis in 1936 [3]. Since these initial studies, much has been learned about the control of oogenesis [4], oocyte maturation [5], oocyte-granulosa cell interactions [6], and cellular organization of the oocyte [7]. Oocyte meiotic maturation is a vital process required for oocyte development. During this process, the LH surge releases oocytes from meiotic prophase arrest and induces resumption of oocyte meiosis and completion of the first meiotic division [8]. This process is initiated when an LH signal is generated in the ovarian follicle. LH binds the mural granulosa cell LH receptor (LHR), activating a G protein which activates the cAMP system. Now, we know that this LH signal targets proteins in both the follicle compartment and the oocyte that regulate oocyte meiotic maturation. The primary targets of the LH signal in the ovarian follicle compartment are the CNP/NPR2 system, the EGF network, and gap junctions [9, 10]. The primary target of the LH signal in the oocyte is the maturation promoting factor (MPF) [11]. Activation of the MPF phosphorylates the SAC, APC/C systems, and other downstream proteins which induce progression of meiosis, namely germinal vesicle breakdown, chromosome condensation, and chromosome segregation. These findings were made in animal models. The cell biology of human oocyte meiotic maturation is less clear.

**Fig. 1:**
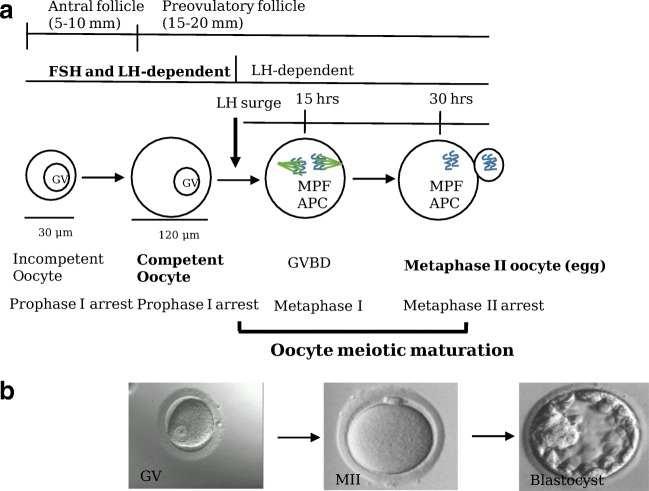
Human folliculogenesis, oogenesis, and oocyte meiotic maturation. **a** Gonadotropins regulate folliculogenesis, oogenesis, oocyte meiotic maturation, and oocyte competence. The first visible sign of meiotic progression is oocyte germinal vesicle breakdown (GVBD) followed by expulsion of the first polar body. The mid-cycle LH surge activates the oocyte maturation promoting factor (MPF) which initiates resumption of meiosis. The MPF activates the oocyte anaphase-promoting complex (APC) which promotes completion of the first meiotic division. MII oocytes remain arrested in metaphase II until fertilization induces completion of the second meiotic division. Oocyte meiotic maturation begins with the LH surge and ends at metaphase II. Competent oocytes support the subsequent development of blastocysts. **b** Human germinal vesicle (GV), MII oocyte (MII), and blastocyst

Oocyte meiotic maturation begins with the mid-cycle LH surge and ends with the formation of a mature oocyte just prior to ovulation (Fig. 1) [12]. The cardinal feature of oocyte meiotic maturation is the formation of a metaphase II–arrested haploid oocyte. LH initiates oocyte meiotic maturation. The induction of oocyte maturation by pituitary gonadotropins was first demonstrated by Heilbrunn in 1939 in frogs. Oocyte maturation begins with the conversion of germinal vesicle (GV) oocytes to MI oocytes then to MII oocytes (Fig. 1). GV oocytes are arrested in prophase I, and may be arrested for up to 50 years in women. The first visible sign of oocyte meiotic maturation is breakdown of the oocyte nuclear membrane referred to as germinal vesicle breakdown (GVBD). This is followed by chromosome condensation and alignment of the chromosomes at the metaphase plate at metaphase I. These oocytes are referred to as metaphase I (MI) oocytes. This is followed by the first oocyte meiotic division, extrusion of the first polar body and formation of a secondary oocyte (mature egg) that arrests at metaphase II until fertilization when the second meiotic division is completed. The molecular mechanisms underlying oocyte meiotic maturation have recently been identified in animals.

LH triggers an explosion of molecular activity in follicle somatic cells [10, 12, 13]. This activates the oocyte maturation promoting factor (MPF) which, in turn, initiates oocyte chromosome segregation. The genesis of the LH signal is the activation of G protein–coupled receptors in mural granulosa cells by the mid-cycle LH surge causing a cAMP spike in the follicular compartment [9, 14, 15]. This rapidly (20 min) suppresses CNP [16] and NPR2 [17], activates the EGF network, and closes gap junctions. The result is reduced oocyte cGMP levels, activation of phosphodiesterase 3A (PDE3A), reduction of oocyte cAMP levels, activation of CDK1 which initiates resumption of meiosis I, followed by chromosome segregation, completion of the first meiotic division, and the formation of an MII oocyte [11, 18]. The formation of a MII oocyte indicates the completion of final oocyte meiotic maturation which is required for the acquisition of oocyte developmental competence.

Most human oocytes retrieved during in vitro fertilization (IVF) are not developmentally competent to form a viable blastocyst [19, 20]. It is important to understand how oocytes acquire developmental competence also referred to as oocyte quality during oogenesis since this is the primary factor responsible for reproductive success. A developmentally competent oocyte is able to develop a mature oocyte, fertilize, cleave, form a blastocyst, implant, and develop into a normal fetus. Oocyte quality is acquired during the process of oogenesis which begins in fetal development during the formation of primordial germ cells and primary oocytes, and ends during final oocyte maturation and completion of the second meiotic division (Fig. 1) [12]. Attainment of oocyte developmental competence requires completion of oocyte cytoplasmic and meiotic maturation [21]. Many cellular processes are responsible for oocyte competence; the major genes responsible for oocyte quality are not yet known [5, 7, 22].

Very little research has been devoted to the human oocyte due to the lack of available human oocytes for research. Most human oocyte research has occurred in the last 40 years with the more readily available supply of human oocytes from the development of human IVF. Most human oocyte studies obtain research oocytes from IVF clinics. The number of human oocyte publications is still limited, as are reviews on LH signaling in human oocyte meiotic maturation [5, 23, 24].

The purpose of this paper is to provide an updated review on this topic. We found 89 human studies in the literature that identified 24 LH signaling pathway proteins involved in human oocyte meiotic maturation (Table 1). These studies show that these ovarian follicle signaling proteins and oocyte cell cycle proteins not only regulate animal and human oocyte meiotic maturation, but also oocyte competence and embryo quality. In addition, we review studies that demonstrate that human oocyte and embryo quality can be improved by manipulating the LH signaling pathway (Table 2). Experimental human in vitro maturation (IVM) studies that incorporate a prematuration culture (PMC) interval manipulated to maintain high cAMP levels by treating with cAMP phosphodiesterase inhibitors or adenylate cyclase stimulators or supplementing with LH signaling pathway molecules; i.e., AREG improves human oocyte competence and embryo quality [101]. This knowledge has helped to improve clinical human IVM efficiency which now is approaching standard IVF efficiency.

**Table 1:** LH signaling proteins regulate human oocyte meiotic maturation

Follicle/oocyte protein	Protein type	Reference(s)
Follicle granulosa cell proteins		
1. LH receptor	G protein–coupled receptor	[25–33]
2. Adenylate cyclase 7 and 9	Enzyme	[30]
3. CNP	Natriuretic peptide	[16, 34, 35]
4. EGF	Growth factor	[35–40]
5. AREG	Growth factor	[33, 35, 41–46]
Follicle cumulus cell proteins		
6. NPR2	Guanylate cyclase	[34]
7. EGF receptor (eRB1)	Tyrosine kinase receptor	[35, 38]
8. Cx43	Channel	[47–55]
9. BMPRII	Serine/threonine kinase	[29, 56, 57]
10. SMAD2/3	Transcription factor	[56, 58–60]
Oocyte-specific factors		
11. GDF9	Growth factor	[25, 61–71]
12. BMP15	Growth factor	[56, 57, 72–77]
Oocyte signaling proteins		
13. GPR3	G protein–coupled receptor	[78]
14. AC3	Enzyme	[78]
15. PDE3A	Enzyme	[78]
Oocyte MPF complex (cell cycle control)		
16. CDK1	Serine/threonine kinase	[25, 79–81]
17. Cyclin B1	Cyclin	[81, 82]
18. WEE1B	Serine/threonine kinase	[80, 83]
19. CDC25	Phosphatase	[25, 84]
Oocyte SAC (cell cycle control)		
20. Bub1, BubR1, Bub3	Serine/threonine kinase	[25, 80–82, 85–87]
21. CDC20	Heterotrimeric G protein	[82]
Chromosome segregation (cell cycle control)		
22. APC (ANAPC1, 4, and 11)	Ubiquitin ligase	[81, 82, 88]
23. Securin-separase	Protease	[81, 82, 88]
24. Cohesin (SMC1, REC8, STAG3)	ATPases	[81, 89–92]

**Table 2:** Effect of IVM/PMC on human oocyte and embryo quality

	Groups	MII	FR	CR	BR
cAMP-modulated IVM systems					
ND, [93]	Conventional IVM	46%^a^	60%		
2006	PMC-PDE-I	67%^a^	58%		
SYM, [94]	Conventional IVM	55.6%	52%^a^	16.7%	5%
2008	PMC-PDE-I	59.7%	65%	20.8%	8.7%
	PMC-Forskolin	62.8%	67%	15.4%	10.2%
	PMC-PDE-I + forskolin	65.4%	76.3%^a^	23.5%	17.6%
VL, [95]	Conventional IVM	60.6%^a^	55.0%^a^	27.3%^a^	
2009	PMC-ECM, PDE-I	81.6%^a^	67.5%^a^	55.6%^a^	
SC, [96] 2015	PMC-PDE-I	50.2%	68.3%	30.5%	
Novel IVM systems					
GPT, [97]	Cumulus-denuded				
1998	wo EGF	33.9%^b^	53.8%	42.8%	
	w EGF	64.3%^b^	72.7%	50%	
	Cumulus-intact				
	wo EGF	79%	45.6%^a^	95.2%	
	w EGF	81%	71.7%^a^	84.8%	
BAI, [98]	Conventional IVM	36.5.0%^a^	73.6%	85.7%	
2011	IVM-EGF/AREG	75.5%^a^	71.8%	85.7%	
SF, [99]	Conventional IVM	48%^c^	31%	23%^c^	8%^b^
2017	PMC-CNP + IVM/ARE	70%^c^	53%	43%^c^	18%^b^
MA, [100]	Standard-IVM	42%^c^	76%	38%^c^	40%
2018	AFF-IVM	34%^c^	73%	36%^c^	25%^c^
	HFF-IVM	59%	80%	60%	42%
	HFF-CGC-IVM	79%^c^	92%	71%^c^	65%^c^

*PMC*, prematuration culture; *MII*, metaphase II; *FR*, fertilization rate; *CR*, cleavage rate; *BR*, blastocyst rate; *PDE-I*, phosphodiesterase inhibitor; *ECM*, extracellular matrix; *w*, with; *wo*, without; *CNP*, C-natriuretic peptide; *P* < 0.05^a^, *P* < 0.01^b^, *P* < 0.001^c^

## Follicle and Oocyte Development

The functional unit of the ovary is the follicle. The primary function of the follicle is to support the development of a competent mature oocyte. The follicle contains a single oocyte surrounded by granulosa cells. Follicular growth and development are complex processes that begin in fetal development and end in about 50 years in most women [1]. The oocyte originates from oogonia which arise from primordial germ cells which first appear in the yolk sac around the third week of gestation. Primordial germ cells migrate to the genital ridge around the fifth week of gestation where they divide by mitosis forming approximately 7 million oogonia during the fifth month of gestation. Primary oocytes form from oogonia when they divide by meiosis. Primary oocytes that are surrounded by a single layer of spindle-shaped cells, the precursors of granulosa cells (GCs), are called primordial follicles which appear during the fifth month of gestation in humans. The spindle-shaped cells differentiate into granulosa cells which proliferate transforming the primordial follicle into a primary follicle. A degenerative process called atresia reduces the number of oocytes from seven million to one million at birth to 500,000 at menarche. Primary oocytes, primordial follicles, and primary follicles remain arrested in the diplotene stage of prophase I until puberty when the ovarian cycle begins.

Much of our understanding of follicular development comes from studies of the rodent 4-day estrus cycle. Pedersen described five follicle stages in the mouse ovary: primordial, primary, secondary (preantral), tertiary (antral), and preovulatory (Graafian) [102]. Primordial follicles continuously leave the non-growing oocyte pool starting at puberty. The conversion of dormant primordial follicles to growing primary follicles is a critical step in folliculogenesis. Primary follicles are composed of cuboidal granulosa cells, a basal lamina, and a 20-μm-diameter primary oocyte. The nature of the converting signal is not yet known [103, 104]. Primary follicles are converted to secondary follicles, and these are composed of two layers of GCs, a zona pellucida, and theca cells. Secondary follicles produce estrogen, progesterone, and androgens and express gap junctions.

The tertiary follicle or antral follicle develops a space filled with follicular fluid called an antrum, which grows reaching a diameter of 2–5 mm. At this stage, 2 million follicle somatic cells, mural granulosa cells (mGCs) and cumulus cells (CCs), surround the oocyte. The theca interna and externa are formed, LH receptors appear, and estrogen becomes the dominant steroid hormone of the follicle as a result of increased follicular steroidogenesis activity. Antral follicle growth is dependent on follicle-stimulating hormone (FSH) and luteinizing hormone (LH). The preovulatory follicle mean diameter is 20 mm [18–24, 101], and mean follicular volume is 3.8 ml (3.1–8.2). The oocyte at this stage attains a maximum diameter of 70 μm.

The ovarian cycle refers to three reproductive processes: folliculogenesis, ovulation, and formation of the corpus luteum. Folliculogenesis, which is highly regulated, refers to the process of ovarian follicle growth and differentiation that primarily occurs during the menstrual cycle. Gougeon described five stages of human follicle development based on follicular size and granulosa cell numbers: primordial follicles, primary follicles, secondary follicles, antral follicles, and preovulatory follicles [105, 106]. The primordial follicle is surrounded by a single layer of pre-granulosa cells, and it has a mean diameter of 30 μm. They appear in the fetus at 16 weeks gestation. At this stage, follicular growth is gonadotropin independent. Primary follicles contain a single layer of cuboidal GCs, they develop a zona pellucida, and oocyte gap junctions (GJs) appear. They appear at 20 weeks gestation.

In the next stages of maturation, follicle growth becomes gonadotropin dependent. GCs proliferate increasing to 600, and the oocyte grows resulting in a secondary follicle. At this point, the follicle diameter is 120 μm, the oocyte diameter increases to 80 μm, and the germinal vesicle (GV) diameter is 25 μm. FSH, LH, androgen, and estrogen receptors appear. Theca cells and GJs appear. In the next phase (class 4), follicles grow to a diameter of 2 mm, GC proliferation increases to 370,000, and the antrum appears. This follicle is called a Graafian follicle. The oocyte now has an eccentric position and is surrounded by multiple layers of GC which are called cumulus cells (CCs).

Follicular maturation refers to the formation of the preovulatory follicle from the tertiary follicle which occurs during the 15 days of the follicular phase [106]. In this phase, the antral follicle becomes a preovulatory follicle or dominant follicle, follicle growth becomes exponential, and the oocyte acquires full developmental competence. The follicle size increases from 5 to 20 mm, GC numbers increase from 370,000 to 50 million, and the oocyte diameter increases to 120 μm. Growth of the dominant follicle is stimulated by FSH, estrogen production increases, the antrum grows, and LH receptors appear.

Oocyte capacitation and final oocyte maturation occur during the first 15 days (follicular phase) of the menstrual cycle [107]. The oocyte reaches its full developmental capacity referred to as full developmental competence during the preovulatory stage prior to the LH surge (Fig. 1). The acquisition of full competence is referred to as oocyte capacitation which is primarily FSH dependent. Oocyte competence is correlated to follicle diameter. Preovulatory follicles with 15–25-mm diameters generally contain competent oocytes. At this point, the oocyte is ready to undergo final oocyte meiotic maturation. The mid-cycle LH surge releases oocytes from prophase arrest and induces chromosome segregation, completion of the first meiotic division, extrusion of the first polar body, and ovulation of the secondary oocyte into the fallopian tube.

The terms recruitment, selection, and dominance describe ovarian follicle maturation during the follicular phase of the menstrual cycle [107]. In the early follicular phase, a cohort of primordial follicles matures while many others degenerate by atresia. This pattern of follicle growth and atresia has been referred to as the “trajectory of follicle growth”. The term recruitment refers to the process by which a follicle cohort enters the growth trajectory. Selection refers to the process that reduces the recruited follicle cohort to one follicle. Selection occurs in the early follicular phase*.* Dominance refers to the one follicle being selected to ovulate [108]. It becomes dominant 7 days before ovulation. Estradiol production increases and becomes the primary steroid in dominant follicles. Estradiol levels are different in the ovarian veins by days 5 to 7 of the cycle [109]. Intrafollicular estradiol levels peak in the dominant follicle in the late follicular phase. This is followed by the mid-cycle LH surge. At the beginning of the LH surge, intrafollicular E2 levels decrease, and progesterone levels increase which reflects GC luteinization [110]. In women, the mid-cycle LH surge triggers GVBD, cumulus cell expansion, and extrusion of the first polar body at 15, 22, and 35 h after the start of the LH surge, respectively (Fig. 1) [111].

## Luteinizing Hormone Receptor

### Mid-cycle Luteinizing Hormone Surge

The menstrual cycle is under neuroendocrine control. Luteinizing hormone (LH) is a member of the pituitary glycoprotein hormone family which consists of LH, FSH, HCG, and TSH. Each is a heterodimer glycoprotein composed of two non-covalently bound polypeptide subunits. They each contain an identical alpha subunit and a hormone-specific beta subunit. The human LHβ, FSHβ, and hCGβ subunits are composed of 121, 110, and 145 amino acids, respectively. The human common α subunit is composed of 92 amino acids. In humans, the LH beta subunit and hCG gene are located on chromosome 19, FSH beta is on chromosome 11, and the common alpha is on chromosome 6. Cloning and DNA sequence of the gene encoding the bovine beta FSH chain were determined in 1986 [112]. Both gonadotropins are synthesized and stored in pituitary gonadotrope granules. Both LH and FSH exist within a single gonadotrope population in the anterior pituitary consistent with the combined secretion of LH and FSH at mid-cycle in humans.

The onset of the LH surge occurs on cycle day 15 of the menstrual cycle. The LH surge is characterized by a 10-fold increase in LH levels in the peripheral circulation [113]. The mean duration of the LH surge is 4 days. How serum LH reaches the mural granulosa cells is not clear; however, LH binds the LH receptor, inducing oocyte maturation and ovulation, 36 and 40 h respectively, after the beginning of the LH surge. The mid-cycle LH surge is induced by circulating estrogen. Mean estradiol levels peak at 200 pg/ml at the end of the follicular phase. This rise in circulating estradiol induces the pituitary LH surge. Estrogen induces the LH surge by acting on the pituitary and hypothalamus. Whether the primary action of estrogen is on the pituitary and/or hypothalamus is still not clear.

The pituitary LH surge is controlled by gonadotropin-releasing hormone (GnRH) secreted by hypothalamic GnRH neurons. How the brain controls the pituitary gland and pituitary gonadotropin secretion was not known until fairly recently. Early studies speculated that a neural factor controls reproduction [114]. Guillemin [115] and Schally [116] simultaneously discovered the neural factor, luteinizing hormone–releasing hormone (LHRH), in 1971. This discovery established the field of neuroendocrinology. The Nobel Prize in Medicine was awarded to Guillemin, Schally, and Yaslow in 1977. Yaslow developed the radioimmunoassay (RIA), a method that utilizes radioactive isotopes to measure hormones and other molecules. Insulin was measured for the first time with the RIA method. A GnRH surge was identified in pituitary stalk blood in rats [117] and primates [118] using the RIA method. The mechanisms underlying the GnRH surge are still not known. Estrogen is probably involved. Estrogen induces a GnRH surge in the ewe [119].

The most important feature of the GnRH system is the inherent pulsatility of GnRH neurons. Many years of research have been devoted to this area [120–123]. GnRH neurons are bipolar neuroendocrine cells that are located in the medial basal hypothalamus. In primates, GnRH neuron cell bodies are primarily located in the medial preoptic area of the hypothalamus, while their axons are primarily found in the median eminence [124]. GnRH is a decapeptide that is stored in GnRH neuron vesicles. The vesicles are transported to the GnRH neuron axon terminals where GnRH is released in a pulsatile fashion into the portal vessels that surround the pituitary gonadotropes. GnRH pulses, in the portal vessels, occur every 30 min in rats [125] and every 60 min in primates. The neural mechanism that controls pulsatile GnRH secretion is still not clear [123]. GnRH neuron excitation-secretion coupling may be involved. Isolated GnRH neurons in vitro release GnRH in a pulsatile fashion [126]. GnRH neurons in vivo generate periodic electrical bursts [127]. Estrogen [128, 129] is probably involved, and GnRH neuron ion channels [130, 131] may have a role. Secreted GnRH binds the GnRH receptors on the pituitary gonadotropes which stimulates cAMP production. This results in increased intracellular calcium which causes the release of LH and FSH. LH and FSH are released into the peripheral circulation in a pulsatile fashion in sheep and rats [132, 133], primates [134], women [135, 136], and men [137]. LH is transported to the ovary where it binds mural granulosa cell LH receptors.

### LH Receptor

The mid-cycle LH surge in humans and animals activates the luteinizing hormone receptor (LHR) also referred to as the luteinizing hormone/choriogonadotropin receptor (LHCGR). LHR is primarily expressed in the mural granulosa cells of the ovarian follicle. The biological actions of LH, required for oocyte maturation, ovulation, and corpus luteal function, in the ovarian follicle are mediated by LHR which is coupled to Gs, the G protein that activates adenylate cyclase and cAMP. This results in an elevation of follicle cAMP levels which affects multiple follicle LH signaling pathway molecules that ultimately activate the maturation promoting factor (MPF) in the oocyte which induces oocyte maturation, resumption of meiosis, and the first meiotic division.

LH receptors belong to the rhodopsin/β2-adrenergic receptor subfamily A of G protein–coupled receptors (GPCR). The LH receptor is a seven-transmembrane domain cell surface protein [138–141]. The human LH/hCG receptor was cloned in 1995 [142]. It is composed of 701 amino acids, 333 amino acids form the seven transmembrane domain segments, and 341 amino acids form the large extracellular domain. The extracellular domain is the hormone-binding domain. LH binds the LH receptor extracellular domain causing a conformational change in G_s_α resulting in replacement of GDP with GTP. The GTPα unit activates adenylate cyclase which converts ATP to cAMP [143–145]. LH receptors are expressed exclusively in mural granulosa cells (GCs), and almost no expression is found in cumulus cells (CCs) or the oocyte in rats and mice [146–148].

LH receptor mRNA expression in mural granulosa cells is regulated during the ovarian cycle. Peng et al. found that LH receptor expression is up and downregulated during the ovarian cycle [149]. In unstimulated rats, LH receptor mRNA expression is not detectable in GC and cumulus granulosa cells. In human menopausal gonadotropin (HMG)–stimulated rats, LH receptor mRNA expression is highest in mural GC in preovulatory follicles and is undetectable in CCs. Post-HCG, LH receptor expression in mural granulosa cells (mGCs) decreases markedly and again increases in the corpus luteum. The LH surge paradoxically downregulates LH receptor expression in preovulatory follicles [150–155].

The LH surge activates mGC LH receptors, which results in a dramatic increase in follicle cAMP production. Approximately 10,000 LH receptors are expressed in each rat GC in preovulatory follicles [156]. Activation of LH receptors causes a 200-fold increase in cAMP levels in mural GCs, which is referred to as the preovulatory cAMP spike [157]. The cAMP spike transmits the LH signal to the cumulus cells and the oocyte via multiple signaling pathways.

LHR mutations cause follicle development abnormalities and infertility in animals and humans. LHR mutations in mice cause an increase in antral follicles and they lack preovulatory follicles which results in anovulation and infertility. Lei et al. generated LH receptor knockout mice [158]. They are infertile, the internal and external genitalia are severely underdeveloped, the ovaries are small, and the ovarian follicles arrest at the antral follicle stage. Zang et al. also generated LH receptor knockout mice [159]. The null mice are infertile, the ovaries are small, and the ovarian follicles grow only to the early antral stage. These studies suggest that LH is required for the development of a dominant preovulatory follicle. Ovarian follicle growth and development beyond the early antral stage require some LH activation of the LH receptor.

**Fig. 2:**
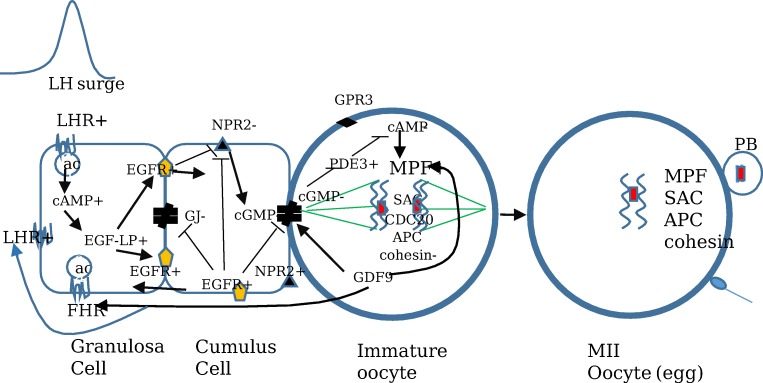
Human model of LH regulation of oocyte meiotic maturation. The LH signal begins with mid-cycle LH activation of the mural granulosa cell LH receptor. The LH signal rapidly suppresses CNP/NPR2, activates the EGF/EGFR network, and inhibits gap junction activity. This reduces follicle and oocyte cGMP levels, activates oocytes phosphodiesterases, reduces oocyte cAMP levels, and activates the oocyte maturation promoting factor (MPF). The MPF initiates resumption of meiosis by protein phosphorylation of downstream proteins. The spindle assembly checkpoint (SAC) proteins are activated at the kinetochore to induce spindle formation and alignment. CDC20 activates the anaphase-promoting complex (APC) which initiates the transition from metaphase to anaphase by degrading securin releasing separase which degrades cohesin. This frees chromosomes to segregate to opposite poles. The first meiotic division is completed and the mature metaphase II oocyte remains arrested until fertilization. +, activation or stimulation; −, inhibition

In humans, LHR is primarily expressed in ovarian follicle granulosa cells [25]. Similar to animals, LH receptor expression is highest in mGCs in preovulatory follicles. LH receptor GC mRNA expression is 10-fold higher in preovulatory follicles compared with small antral follicles [26, 27]. LHR expression is suppressed by the LH surge. The LH surge downregulates GC LH receptor expression in preovulatory follicles in women [28, 29]. The mid-cycle LH surge activates mural GC LH receptors which activates adenylate cyclase [30] and increases follicle cAMP levels (Fig. 2).

Mutations of LHR cause ovarian follicle development abnormalities and infertility in women. Women with LHR mutations experience amenorrhea, lack preovulatory follicles, do not respond to exogenous HCG, and are infertile [160]. Toledo et al. in 1996 reported a LHR mutation case in a 21-year-old with 46XX primary amenorrhea, normal secondary sexual development, and infertility [161]. Ovary size was normal on ultrasound. Histologic analysis of an ovarian biopsy revealed primordial follicles, preantral follicles, and antral follicles. No preovulatory follicles were seen. DNA analysis of the LHR gene revealed a single nucleotide change (guanine to cytidine at position 1787). This caused a substitution of a proline for an alanine in the LH receptor. Human embryonic kidney cells, transfected with the mutant LH receptor gene, did not respond to HCG.

Latronica et al. reported a 40-year-old with irregular menstrual cycles, normal menarche and pubarche, and infertility [162]. The ovaries were normal size on pelvic ultrasound, the FSH level ranged from 5 to 17 IU/l, the serum estradiol level was < 50 pg/ml, total testosterone was normal, and karyotype was 46XX. A six-nucleotide deletion in the LH receptor gene resulted in deletion of leucine and valine. Cells transfected with the mutated LHR cDNA revealed reduced LHR expression and reduced (1.5- vs. 30-fold in wild-type transfected cells) stimulation of cAMP in response to HCG. These studies show that LHR mutations cause anovulation and infertility. These studies suggest that human early follicle development is primarily FSH dependent, while preovulatory follicle development requires LH. LHR may be abnormal in PCOS. LHR mRNA is overexpressed in GC and theca cells in women with PCOS when compared with normal controls [163].

The LHR mRNA expression in cumulus cells (CCs) may predict oocyte quality. Yang et al. studied LHR mRNA expression in cumulus cells from 35 PCOS women who underwent 50 IVM cycles [31]. Patients were given HCG 10,000 IU on cycle days 7 and 13, and oocytes were retrieved 36 h post-HCG. Cumulus-oocyte complexes (COCs) were separated into 3 groups: dispersed CCs (group A), compacted CCs (group B), and sparse CCs (group C). Dispersed CCs were enclosed by an expanded CC and 1–2 corona cell layers, compacted CCs contained 4–5 corona cell layers, and sparse CCs had few coronal cells. COCs were cultured in IVM media supplemented with human follicular fluid (FF), FSH, HCG, and EGF for 24, 48, and 72 h. MII oocytes were inseminated by ICSI; embryos were grown to days 5 and 6. IVM cycles resulted in embryo transfers on day 6. CCs were collected from the GV-staged oocytes, mRNA was extracted, and RT-PCR for LHR, FSH receptor, and EGF receptor was performed. The MII rate (Grp A-24h-70%, 48h-80%, 74h-85%), blastocyst rate (A-40%, B-23%, C-23%), and CC LH receptor mRNA expression levels were higher in group A than groups B and C. The study concluded that oocytes from expanded/dispersed CCs with high CC LH receptor mRNA expression levels have better oocyte quality compared with oocytes from unexpanded CCs with low LHR mRNA levels.

Regan et al. studied LHR mRNA expression density in 327 ovarian follicles from young and old patients treated with IVF [29]. Granulosa cell LH receptor density was measured by immunofluorescence from GCs retrieved after standard controlled ovarian hyperstimulation. GC LHR density was increased in young women compared with older women. Higher live birth rates were found in young women with high GC LHR density compared with older women with lower GC LHR density. They also found that the LH surge–induced downregulation of the LH receptor was evident mostly in the larger follicles in young women. LHR downregulation was not observed in follicles from older women. This suggested to the authors that large follicles are more receptive to the LH surge than smaller follicles since they downregulated appropriately. This may indicate a GC dysfunction in small follicles and follicles in older women. Also, the FSH dose used for IVF stimulation was not associated with GC LHR expression levels which suggests that other factors other than gonadotropins regulate GC LHR expression during follicular development. The authors concluded that high GC LH receptor density and normal downregulation of the GC LH receptor by the LH surge which is primarily found in preovulatory dominant follicles are associated with oocyte quality.

Maman et al. found higher CC LHR mRNA expression in MII oocytes compared with MI and GV oocytes; however, higher LHR expression was not associated with higher fertilization rates [32]. Huang et al. found that LHR CC mRNA expression was not associated with a higher pregnancy rate [33]. Whether high or low LHR mRNA expression in CCs is associated with oocyte and embryo quality is not clear.

## Follicle C-natriuretic Peptide and Natriuretic Peptide Receptor 2

The first target of the LH signal in the follicle compartment is the CNP/NPR2 system. LH suppresses the CNP/NPR2 system and within minutes reduces cGMP follicle levels. This ultimately leads to activation of the oocyte maturation promoting factor (MPF) which initiates resumption of meiosis and chromosome segregation. The CNP/NPR2 system is the major inhibitor of oocyte meiosis progression in the ovarian follicle. The first clue that ovarian follicle somatic cells express an inhibitor that prevents meiotic progression came when Pincus and Enzman in 1935 observed spontaneous oocyte maturation within 1–2 h in vitro at the time oocytes were separated from ovarian follicle somatic cells [164]. This phenomenon occurs in mouse, sheep, cow, pig, monkey, and human oocytes [165].

Initial studies suggested that the follicle factor responsible for oocyte meiotic arrest was cAMP [166–168]. Later studies showed that cAMP produced by the oocyte, not cAMP from the follicle, was the major inhibitor of oocyte meiotic arrest. Mehlmann et al. injected mouse oocytes with antibodies against stimulatory G protein (Gs) which stimulates oocyte adenylyl cyclase and cAMP production. This caused resumption of meiosis, 80% of the injected oocytes developed GVBD showing that oocyte Gs is required for meiotic arrest [169]. Horner et al. showed that oocyte GPR3 activates oocyte adenylyl cyclase (AC3) which produces cAMP in the oocyte [170]. Later, Mehlmann et al. showed that the oocyte Gs is linked to the oocyte receptor G protein–coupled receptor 3 (GPR3) which is required for meiotic arrest in mice [171] and humans [78].

cGMP is the major factor in the follicle responsible for oocyte meiotic arrest [9]. Norris et al. found that reducing oocyte cGMP levels increased the activity of oocyte phosphodiesterase 3A (PDE3A) and lowered levels of oocyte cAMP which induced resumption of meiosis [172]. They also found that blocking follicle gap junctions reduced oocyte cGMP. They concluded that cGMP produced by ovarian follicle somatic cells enters the oocyte through gap junctions and inhibits PDE3A activity which allows high levels of cAMP to accumulate in the oocyte. High oocyte cAMP levels trigger resumption of meiosis. How is cGMP produced in the ovarian follicle compartment?

C-natriuretic peptide (CNP), also called natriuretic peptide precursor C (NPPC), and its receptor guanylyl cyclase natriuretic peptide receptor 2 (NPR2) produce cGMP in the ovarian follicle compartment. CNP and NPR2 are highly expressed and regulated in ovarian follicles during the rat estrus cycle [173]. In 2010, Zhang et al. showed that CNP mRNA expression was 10-fold higher in mural GCs compared with CCs, and NPR2 mRNA expression was 2-fold higher in CCs compared with mGCs [174]. CNP increased oocyte cGMP levels in the follicle which inhibited meiotic resumption. They also studied the role of oocyte-secreted factors (OSFs) on the follicular compartment. They found that bone morphogenetic peptide 15 (BMP15) combined with growth differentiation factor 9 (GDF9) increased CC NPR2 mRNA expression. This suggested that BMP15 and GDF9 primarily inhibit meiotic progression.

Based on these findings, the authors proposed a model for oocyte meiotic arrest. Mural GC CNP activates CC NPR2 which increases cGMP production in the follicular compartment. Follicle cGMP diffuses through follicle/oocyte gap junctions into the oocyte. Oocyte cGMP inhibits oocyte PDE3A activity which increases oocyte cAMP. High oocyte cAMP levels inhibit resumption of meiosis. Genetic studies support this model. NPR2 mutant mice are infertile due to premature resumption of meiosis caused by a lack of follicle cell cGMP production which results in oocyte fragmentation and poor embryo development [175, 176]. Humans with NPR2 mutations develop acromesomelic dysplasia, Marateaux type (AMDM) [177]. Infertility has not been described in these patients.

LH exposure inhibits the CNP/NPR2 system which induces oocyte meiotic resumption in preovulatory follicles. LH reduces cGMP levels very rapidly in the mural GC, CC, and oocyte. Time-lapse recordings of cGMP levels in mouse follicles showed a decrease in cGMP levels in mural GCs within 1 min of LH exposure, in CC within 5 min, and in oocytes within 10 min [178]. LH reduced NPR2 activity in mural GCs and CCs within 3 h of LH exposure by dephosphorylation, and NPR2 protein levels did not change. LH also reduced CNP levels within 2 h of LH exposure [17]. How the LH deactivates NPR2 is not clear. One possible mechanism is that LH activates EGF/EGFR which inhibits NPR2. EGF receptor was activated within 15 min after LH application [179] and resulted in reduced follicle cGMP levels [180].

In humans, the ovarian follicle CNP/NPR2 system has not been well studied. One paper showed that LH reduces CNP levels in human ovarian follicular fluid [16], and one paper found NPR2 mRNA expression in human mural GCs [34] (Fig. 2).

## Epidermal Growth Factor Network

The second target of the LH signal is the ovarian follicle EGF network. The activated EGF network transmits the LH signal from the mural granulosa cells (mGCs) to the oocyte in preovulatory follicles. The activated EGF network inhibits the CNP/NPR2 system and gap junctions reducing follicle and oocyte cGMP. This occurs via the EGF receptor which is highly expressed in follicle cumulus cells. cGMP is the molecule that transmits the LH signal from the follicle to the oocyte.

EGF-like protein (EGF-LP) growth factors stimulate oocyte meiotic maturation and cumulus cell expansion [36, 181]. The first evidence that EGF regulates these processes came in the 1980s [182, 183]. Recently, Park et al. showed that LH, within 3 h, stimulates production of epidermal growth factor-like protein (EGF-LP) family members: amphiregulin (AREG), epiregulin (EPI), and beta-cellulin (BTC) in mice ovaries [184]. EGF-LP expression was restricted to mural GCs in preovulatory follicles. EGF-LP stimulated oocyte meiotic maturation within 4 h of exposure to AREG and EPI as GVBD was observed in 100% of the oocytes in preovulatory follicles. EGF-LP also stimulated cumulus cell expansion within 8 to 12 h of exposure to AREG, EPI, or BTC. Cumulus cell expansion genes were upregulated. Hyaluronan synthase 2 (HAS2), prostaglandin-endoperoxide synthase 2 (Ptgs2), and tumor necrosis factor alpha–induced protein 6 (Tnfaip) mRNA expression levels increased within 3 h of exposure to EPI, AREG, and BTC. LH stimulates production of AREG, EPI, and BTC via p38 mitogen–activated protein kinase (MAPK) [185, 186]. In null AREG and EREG mice, meiotic resumption is reduced [187].

Activation of the EGF receptor (EGFR) is required for oocyte meiotic resumption [188] and cumulus cell expansion. EGF receptors are members of the tyrosine kinase receptor (TKR) family that includes ErbB1, ErbB2, ErbB3, and ErbB4 [189]. EGF tyrosine kinase receptors are single-transmembrane domain receptors that regulate cellular proliferation. EGF binding sites were first identified on rat GCs in secondary ovarian follicles in 1986 [190]. The highest EGFR mRNA expression levels are in CCs [181]. LH causes EGF receptor (EGFR) phosphorylation within 3 h of exposure [184]. EGFR activation is required for LH action. EGFR inhibitors block LH-induced oocyte meiotic resumption and CC expansion. EGFR signals via extracellular signal–regulated kinases 1 and 2 (ERK1/2) [191, 192]. Granulosa cell–specific EGFR null mice fail to resume meiosis [193].

The LH-activated EGF network targets the CNP/NPR2 system and follicle/oocyte gap junctions. The EGF network inhibits CNP/NPR2 production of cGMP and suppresses gap junction activity. LH-activated EGFR reduces CNP mRNA expression levels in granulosa cells [176], and cGMP levels in the follicle and oocyte. Reduced oocyte cGMP levels activate oocyte PDE which reduces oocyte cAMP levels. This activates the oocyte maturation promoting factor (MPF) which initiates resumption of meiosis [180, 194].

The EGF system is found in the human follicle [36]. The human primordial follicle does not express EGF or EGFR [37]. Maximum EGF and EGFR expression occurs in preovulatory follicles [38]. EGF is expressed in human preovulatory follicle follicular fluid (FF) [39]. Reeka et al. failed to detect EGF in human FF, but found EGF in GCs [40]. Zamah et al. found EGFR mRNA expression in human GCs from IVF patients [35].

LH increases production of EGF-LP in human GCs and CCs (Fig. 2) [41, 42]. AREG mRNA and protein are expressed in human GCs [41]. AREG is the most abundant EGF-like growth factor in follicular fluid aspirated at oocyte retrieval in IVF patients stimulated with gonadotropins. Rimon et al. reported a 286-fold increase in AREG expression in GC from IVF patients [43]. Whether EGF-LP suppresses CNP/NPR2 and inhibits gap junctions resulting in oocyte meiotic resumption is not known. One study found that LH reduces CNP levels in human FF [35].

Mixed results have been found in studies investigating the association between EGF molecules and oocyte quality. Feuerstein et al. found a positive correlation between CC AREG mRNA expression and blastocyst rate [44]. Huang et al. found that high CC AREG mRNA expression from MII oocytes was associated with pregnancy rate [33]. Zamah et al. found that AREG levels from FF correlated with oocyte maturation rate [35]. Hoffman et al. found that human EGF FF levels were inversely correlated with oocyte maturation [45]. Inoue et al. found that FF AREG levels were inversely correlated to fertilization rate and was not correlated with embryo quality [46]. A reliable EGF network oocyte quality biomarker has not been identified.

## Gap Junction Communication

The third major target of the LH signal is the follicle/oocyte gap junction. Gap junction channels allow direct communication between cells. They allow ions and molecules to pass from the cytoplasm of one cell to the cytoplasm of the other, thereby coupling the cells metabolically and electrically. Studies have demonstrated that small fluorescent dye molecules injected into one cell can pass into adjacent cells, provided the molecules are smaller than 1000 Da. This suggests a gap junction channel diameter of 1.5 nm so cells can share small molecules like ions, nucleotides, and amino acids, but not large molecules like proteins or nucleic acids. The molecular mass of cGMP is 345.2 and cAMP 507 Da. Gap junctions are formed from connexons which are formed from connexins. Several distinct connexins have been identified. Connexins are named by their molecular weights. Connexin 43 has a molecular weight of 43 kDa. Gap junction channels behave like conventional gated ion channels. They flip between open and closed states, switching rapidly within seconds. Gap junctions regulate hearing, cardiac and neural function, liver function, and ovarian folliculogenesis and oogenesis [195, 196].

Gap junctions are present between mural granulosa cells and cumulus cells [166], and between cumulus cells and oocytes [197]. Connexins are expressed in ovarian follicles [198, 199]. Connexin 43 and 37 are the primary functional connexins in the ovarian follicle. Cx43 is the major connexin expressed in rat granulosa/cumulus cells [200]. Cx37 is primarily expressed in the oocyte [201]. Gap junctions regulate meiotic arrest and resumption [198]. CNP/NPR2 produces cGMP in cumulus cells which diffuses into oocytes through Cx43 gap junctions which elevates oocyte cGMP. This maintains oocyte meiotic arrest [202].

LH disrupts gap junction (GJ) communication between the follicle somatic cells and oocyte which induces resumption of meiosis. Initial gap junction studies found that loss of CC gap junctions induced GVBD in rat oocytes [203, 204]. LH closes follicle GJs [205, 206] and oocyte GJs [207]. LH inhibits rat gap junction activity via MAPK phosphorylation [207] of Cx43 and also reduces Cx43 protein levels [208]. The activated EGF network also closes cumulus cell and oocyte gap junctions [180]. AREG activates EGF receptor which closes follicle gap junctions. This prevents transport of cGMP from the follicle somatic cells to the oocyte which induces resumption of meiosis [172]. In porcine IVM conditions, LH closes connexin 43 gap junctions [209]. Mice lacking connexin 37 lack mature Graafian follicles, and the oocytes are arrested at the early antral stage, fail to grow, fail to ovulate, and develop inappropriate corpus lutea [210, 211].

Gap junctions regulate human ovarian folliculogenesis (Fig. 2) [47]. Human ovarian follicles express 15 connexins, but only Cx43 and Cx37 form gap junctions [48, 49]. Cx43 is the major gap junction connexin in human cumulus cells [49]. Human gap junctions are regulated by cAMP [50]. BMP15 reduces Cx43 expression and gap junction activity in human granulosa cell lines and granulosa cells [51]. Whether LH closes human follicle/oocyte gap junction activity is not known. We found no studies addressing this question.

Cumulus cell mRNA expression studies suggest Cx43 may be a potential biomarker of human oocyte quality. Feuerstein et al. found that Cx43 was associated with improved oocyte quality [52]. Wang et al. found higher live birth rates in oocytes with high CC Cx43 mRNA levels [49]. Low CC Cx43 expression was found in low responders [53]. Reduced expression of Cx43 at oocyte retrieval was associated with improved oocyte quality [54]. Double trigger with GnRH agonist and HCG reduced Cx43 mRNA CC expression and improved oocyte and embryo quality [55]. It is not clear whether increased or decreased Cx43 CC mRNA 43 expression is associated with oocyte quality.

## Oocyte-Secreted Growth Factors: BMP15 and GDF9

The major oocyte-secreted growth factors studied so far are BMP15 and GDF9. Whether oocyte BMP15 and GDF9 are targets of the LH signal during oocyte meiotic maturation in animals or humans is not known. It is well established that pituitary gonadotropins regulate ovarian folliculogenesis and oocyte quality [212, 213]. Recent work suggests that the oocyte regulates folliculogenesis [214–219] and oocyte quality [220, 221]. Oocyte-secreted factors (OSFs), bone morphogenetic protein 15 (BMP15) and growth differentiation factor 9 (GDF9), are important regulators of ovarian follicle development [222, 223]. They are exclusively expressed in the oocyte, are present in all stages of follicular growth [224], and may have a role in human oocyte maturation. Recently, other OSFs have been identified. Oocyte interleukin-7 (IL-7) was found to possibly have a role in human oocyte maturation [225].

BMP15 and GDF9 regulate animal and human ovarian folliculogenesis. Initial studies, 50 years ago, found that premature luteinization of the rabbit ovarian follicle occurs when the oocyte is removed from the follicle. This suggested that an oocyte-secreted factor (OSF) prevents luteinization. The authors proposed the concept that the oocyte controls follicle somatic cell processes [226]. Mouse genetic studies showed that GDF9 is required for normal ovarian follicle development [227]. GDF9 null mice ovarian follicles do not progress beyond the primary follicle stage, which results in infertility. During the last decade, studies in animals showed that GDF9 and BMP15 are involved in cumulus cell expansion [228, 229] and oocyte quality [72, 221, 230].

BMP15 and GDF9 are members of the transforming growth factor-beta (TGF-β) superfamily, a structurally conserved group of proteins with at least 35 members [231]. The members of the superfamily are classified into subfamilies. They include the TGF-β subfamily (TGF-β1-β3); the bone morphogenetic (BMP) subfamily is the largest with 20 members, the growth differentiation factor (GDF) subfamily with 9 members, the activin/inhibin subfamily, the glial cell–derived neurotrophic factor (GDNF) subfamily, and anti-Mullerian hormone. GDF9 was initially discovered in 1993 [232]. The TGF-β superfamily is composed of growth factors that regulate reproduction, embryo development, and tumor growth [233].

The TGF-β superfamily members act by binding two types of serine/threonine kinase cell surface receptors called types I and II. Seven type I and five type II receptors have been identified. BMP15 and GDF9 bind several receptors including the serine/threonine kinase receptor type II bone morphogenetic receptor type-2 (BMPR2) [234], bone morphogenetic receptor type-IB (BMPR1B) also known as activin receptor–like kinase (ALK6) and bone morphogenetic receptor type-IA (BMPR1A) also known as ALK3. BMP15 and GDF9 primarily bind BMPR1B which is the major TGF-β receptor in ovarian follicles [58, 222, 235, 236]. GDF9 and BMP15 signal through SMAD transcription factors (fusion of *Caenorhabditis elegans* Sma genes and the *Drosophila* Mad, Mothers against decapentaplegic) [237] to regulate granulosa cell function in animals and humans [58].

### GDF9

Growth differentiation factor 9 (GDF9) is an oocyte-derived growth factor [238] required for folliculogenesis and oogenesis. It is a protein in the TGFβ superfamily, composed of 454 amino acids with a molecular weight of 53.4 kDa. GDF9 controls follicle growth by stimulating ovarian follicle granulosa cell proliferation at all stages of follicle development [239, 240]. It stimulates granulosa cell proliferation [241] by both increasing GC FSH receptor expression [242] and preventing GC apoptosis [243].

GDF9 is required for oogenesis. GDF9 null mice are infertile due to severe follicle and oocyte abnormalities [227]. The ovaries are small, and primordial and primary follicles never develop more than only 1 layer of granulosa cells. Follicular development never progresses beyond this early stage. The ability of the granulosa cells to proliferate is severely limited. The primary follicle oocytes are enlarged (70-μm diameter); they resemble antral follicle oocytes. Electron microscopy oocyte studies found perinuclear organelle aggregation, abnormal Golgi complexes, and failure to form cortical granules [244]. This study demonstrated for the first time that the oocyte controls the progression of follicular development.

GDF9 promotes cumulus cell expansion during preovulatory follicle development. LH stimulates CC expansion which is essential for the acquisition of oocyte quality [221]. The factors that control CC expansion are still not known. GDF9 regulates numerous CC functions that are involved in CC expansion [245]. GDF9 induces CC expansion genes including pentraxin (Ptx3), hyaluronan synthase 2 (Has2), tumor necrosis factor alpha–induced protein 6 (Tnfaip6), and prostaglandin-endoperoxide synthase 2 (Ptgs2) [246]. GDF9 also inhibits granulosa cell LH receptor mRNA expression [246]. RNA interference studies in mice decrease oocyte GDF9 protein expression, prevent CC expansion, and reduce Has2 and Pgs2 mRNA expression [247]. In addition, GDF9 regulates CC cholesterol biosynthesis [248] and glycolysis [249] which is required to support the metabolic activity during CC expansion. SMAD mice knockouts demonstrate that SMAD 2, 3, and 4 are required for CC expansion [250, 251]. Recent studies suggest that BMP15:GDF9 mouse and human heterodimers are potent regulators of CC expansion [252]. These studies support the hypothesis that GDF9 regulates CC expansion.

The role of GDF9 in human folliculogenesis, cumulus cell expansion, and oocyte meiotic maturation is not clear. GDF9 is expressed in human oocytes [25, 61, 62]. Aaltonen et al. studied GDF9 expression in ovarian biopsies from women under the age of 35 [61]. They found GDF9 mRNA expression in primary oocytes. Primordial oocytes did not express GDF9. Because they did not find antral follicles or preovulatory follicles in their biopsy specimens, they were not able to study GDF9 in these later stages. GDF9 stimulates human granulosa cell (GC) proliferation [63, 64]. GDF9 stimulates activin signaling [62] and inhibits follistatin [65] in preovulatory luteinized GC from women undergoing IVF. GDF8 downregulates Ptx3, a cumulus cell expansion gene, in human GC [66, 67]. Huang et al. reported the first human study on GDF9 regulation of human-luteinized GC cycle progression [68]. GDF9 upregulates both cyclin D1 and E mRNA and protein via ERK42/44 and SMAD3.

Human genetic studies suggest that GDF9 regulates human folliculogenesis and oocyte development. GDF9 mutations cause premature ovarian insufficiency (POI). Seven GDF9 human mutations have been identified that cause POI [253–256]. GDF9 mutations may cause diminished ovarian reserve [256]. Oocyte GDF9 expression is reduced in PCOS patients [69–71]. This suggests that low GDF9 expression may block antral follicle development, the main follicle abnormality found in PCOS.

GDF9 targets including HAS2, TNFA1P6, PTGS2, and gremlin 1 are potential biomarkers of oocyte quality [246, 257, 258]. In humans, CC HAS2, PTGS2, and gremlin mRNA expression correlates with oocyte quality [259, 260]. Feuerstein et al. found that CC PTGS mRNA is associated with oocyte maturation [44]. Gode et al. found that increased FF GDF9 protein levels correlated with improved oocyte maturation and embryo quality [261]. These studies suggest that CC expansion genes and GDF9 in FF are associated with oocyte quality.

The GDF9 receptor, BMPRII, is also a potential biomarker of oocyte quality. Regan et al. studied granulosa cell BMPR1B mRNA density in young and old women treated with IVF [29]. In young women, no correlation was found between GC BMPR1B density and GC LHR density (*R*^2^ = .078); as expected, GC BMPPR1B density did not increase and was downregulated, as LHR density increased. In older women, BMPR1B density increased as LHR density increased (*R*^2^ = 0.87; *p* = 0.004). The authors concluded that normal downregulation of GC BMPR1B is associated with oocyte quality.

### BMP15

Bone morphogenetic protein (BMP15) is a 392-amino acid dimeric protein in the TGF-β super family, exclusively expressed in the oocyte. BMP15 is expressed in the oocyte throughout follicular development. It binds granulosa cell TGF-β receptors and activates SMAD transcription factors that regulate gene expression. BMP15 stimulates folliculogenesis, cumulus cell expansion, oogenesis, and oocyte maturation and controls ovulation number and oocyte developmental competence. BMP15 human mutations cause ovarian dysgenesis and premature ovarian failure.

BMP15 stimulates proliferation of ovarian follicle GCs [236, 242, 262], stimulates cumulus cell expansion [230, 263], and inhibits ovarian follicle steroidogenesis. In mice, BMP15 regulates CC expansion via SMAD 2/3 stimulation of gene transcription [250, 264, 265], stimulation of CC EGFR expression [266], and inhibition of CC apoptosis [267].

BMP15 promotes oocyte meiotic arrest by increasing follicle cGMP levels by increasing follicle CNP/NPR2 activity, by increasing NPR2 gene expression [268], and by modulating gap junction activity [51]. In preovulatory follicles, BMP15 inhibits FSH receptor expression and reduces progesterone and estrogen production [269].

Sheep with homozygous BMP15 mutations [270] are infertile and develop streak gonads, and folliculogenesis is blocked at the primary follicle stage [271, 272]. Ovulation rates are increased in sheep with heterozygous BMP15 mutations [270].

The role of BMPs in human folliculogenesis and oogenesis is still unclear [273]. Human ovarian follicles express BMPs and BMP receptors. BMP15 protein and mRNA are expressed in oocytes and granulosa cells in fetuses and young women [73]. BMP6 and BMP15 are the most highly expressed BMPs in pre-antral human follicles. GDF9, BMPR2, and SMAD3–4 are also expressed in human pre-antral follicles [56]. BMP2, BMP4, BMP7, BMP15, GDF8, and GDF9 are present in human pre-antral follicle follicular fluid (FF). BMP4, BMP5, BMP6, BMP7, and BMP8 are also expressed in human GCs [74]. BMPRII is the most highly expressed type II receptor in cumulus cells [57]. ALK1 through ALK7 are also expressed primarily in pre-antral and antral follicles [56].

How BMP15 regulates ovarian folliculogenesis and oocyte maturation in humans remains unclear. BMP15 suppresses ovarian follicle steroidogenesis and gap junction activity. BMP15 prevents luteinization by suppressing GC progesterone production. BMP15 and GDF9 decrease GC progesterone production by inhibiting StAR expression [75, 76]. BMP15, BMP7, and BMP4 regulate Cx43 expression [51, 274]. cAMP enhances BMP15-regulated SMAD 1/5/8 signaling in human granulosa cells, suggesting communication between the follicle cAMP system and BMP15/SMAD [59]. BMP15 and GDF9 augment FSH/cAMP stimulation of aromatase mRNA expression in human cumulus cells via SMAD 2/3 [60].

Human BMP15 mutations were first reported in 2004 in two Italian sisters with premature ovarian insufficiency (POI), ovarian dysgenesis, and a granulosa cell proliferation deficiency [275]. Over 1236 cases of POI associated with BMP15 mutations have been described. The risk of a BMP15 mutation is 10× higher in POI patients [276].

BMP15 is a potential biomarker of human oocyte quality. Higher oocyte maturation rates were found in oocytes from follicles with high BMP15 follicular fluid (FF) levels [77, 277]. In IVF poor responders, high BMP15 FF levels are associated with higher fertilization rates, cleavage rate, and good-quality blastocyst compared with low BMP15 FF levels [77]. Li et al. studied 196 infertile women and 2426 COCs. Increased BMP15 and GDF9 mRNA levels in CC correlated with oocyte maturation, fertilization, and embryo quality [72]. Higher oocyte quality was found in oocytes from follicles with high cumulus cell BMP2 expression levels [278]. Dysregulation of activin receptor–like kinase 6 (ALK6) in human GCs is correlated with decreased ovarian reserve [28]. A BMP15 single nucleotide polymorphism is associated with ovarian hyperstimulation [279].

## Oocyte Maturation Promoting Factor: Cyclin-Dependent Kinase 1 and Cyclin B1

The major target of the LH signal in the oocyte is the maturation promoting factor (MPF). The activated MPF initiates resumption of meiosis in the preovulatory follicle. The MPF complex has two components, a cyclin-dependent protein kinase1 (CDK1), also known as cell division cycle protein 2 (Cdc2), and cyclin B [280]. The MPF acts by phosphorylating downstream proteins. Three of the major systems are the SAC proteins, APC/C, and the securing-separase/cohesin proteins. These cell cycle control proteins first appeared 1 billion years ago and have been conserved in all eukaryotic species from yeast to humans. The same cell cycle mechanisms that operate in yeast and invertebrates also operate in humans. Phosphorylation of these proteins by CDK1 induces cell cycle progression and completion of the first and second meiotic divisions. The MPF and the other components of the cell cycle machinery are not well characterized in human oocytes.

The MPF controls both the mitotic and meiotic cell cycles. The cell cycle is the fundamental way by which cells grow and divide. The cell cycle is divided into four distinct phases: M phase, G1 phase, S phase, and G2 phase. During the M phase, the cell divides by mitosis and cytokinesis. The G1 phase is the gap between the M phase and S phase. During the S phase, the DNA replicates. The G2 phase is the gap between the S phase and M phase. The interphase is the period between one M phase and the next M phase. During interphase, the cell grows continuously. The duration of the M phase in an average eukaryotic cell is 1 h. The average cell cycle duration is 24 h. The early embryonic cell cycle duration in preimplantation human embryos is 12–24 h. The cell cycle is controlled by a biochemical machine composed of proteins that duplicate and divide the cell and its contents.

The MPF was purified from *Xenopus* eggs [281, 282] and starfish oocytes [283]. The human CDK1 cDNA [284] and protein sequence [285] were subsequently identified. CDK1 is composed of 297 amino acids. The molecular weight is 34 kDa. The crystal structure of CDK1 was recently reported [286]. CDK1 is a serine/threonine-specific kinase activated by a cyclin partner. The CDK family is composed of 20 proteins divided into two major groups: cell cycle (CDK1, 2, 3, 4, 6, 5, 14, 15, 16, 17, 18) and transcriptional [7–13, 19, 20, and]. The cyclin family is composed of 30 proteins [287, 288]. CDK1 controls the cell cycle. Seventy-five targets of CDK1 have been identified that regulate many aspects of the mitotic cell cycle, including DNA replication and segregation, cell differentiation, cell polarity and morphology, genome stability, transcription, and metabolism [289]. CDK1 also regulates meiosis.

High oocyte cAMP levels prevent resumption of oocyte meiosis. High oocyte cAMP levels are maintained by constitutively active oocyte GPR3 receptors [8] and low oocyte phosphodiesterase (PGE3A) activity maintained by high oocyte cGMP levels which prevents hydrolysis of cAMP to AMP [290]. The phosphodiesterase (PDE) superfamily is composed of 11 PGE gene families [291]. PDE3A is highly expressed in mouse oocytes [292]. Resumption of meiosis is blocked in PDE3A knockout mice [293]. Mehlmann et al. found that oocyte G protein–coupled receptor 3 (GPR3) and GPR12 activate oocyte adenylate cyclase which increases cAMP [169]. GPR3 is essential for meiotic arrest in mice. GPR3 is expressed 14-fold higher in the oocyte compared with follicular somatic cells. GPR3 knockout mice undergo spontaneous oocyte nuclear maturation [171], and mice lacking adenylyl cyclase-AC3 spontaneously resume meiosis progression [170].

High oocyte cAMP levels inhibit the MPF via activation of oocyte phosphatases. The major MPF oocyte phosphatase inhibitors are WEE1B, CDC25, and CDC14A [11, 294]. CDC25A and CDC25B phosphatases inactivate Cdk1/Cyclin B, which maintains oocyte meiosis arrest in mice [295–297]. CDC14A phosphatase also inhibits CDK1 [298]. In vivo knockdown of WEE1B induces spontaneous resumption of meiosis [299, 300].

The LH signal activates the MPF [301]. LH reduces follicular oocyte GMP levels which activates oocyte PDE3A. This in turn reduces oocyte cAMP levels. Low oocyte cAMP levels inactivate oocyte phosphatases, WEE1B and CDC25 [302, 303], which dephosphorylate and activate oocyte CDK1 [18]. The activated CDK1-cyclin B complex phosphorylates many downstream substrates including spindle assembly checkpoint (SAC) proteins localized at chromosome kinetochores [304]. The primary SAC proteins include Mad1, Mad2, Bub1, BubR1, Bub3, Mps1, and aurora B/C kinase. The main function of SAC proteins is to assure that each phase of the cell cycle is completed prior to progressing to the next phase, and to guide microtubule spindle attachment to the kinetochores [305, 306]. SAC protein function is well studied in mitosis while in meiosis their functions are less clear [307].

The primary target of the SAC is the anaphase-promoting complex/cyclosome (APC/C) [308]. The APC/C is an ubiquitin ligase that adds ubiquitins on target proteins which are recognized and degraded by the proteasome. SAC proteins Mad1, Mad2, and BubR1 inhibit the APC/C. SAC proteins inhibit the APC/C from targeting securin (anaphase inhibitor) and cyclin B [304], thereby preventing resumption of meiosis. The activated APC/C-Cdc20 induces entry into anaphase and chromosome segregation [307]. Separase is inactive when bound to securin. At anaphase, the APC/C activated by Cdc20 cleaves securin (anaphase inhibitor) which activates separase. Activated separase cleaves the cohesin complex subunits which induces chromosome segregation.

In humans, the oocyte MPF complex is not well characterized [309]. CDK1 [25, 79, 80], CDC25 [25, 84], and WEE2 are expressed in human oocytes [80]. Sang et al. found WEE2 mutations in four women with fertilization failure [83]. The age of the patients ranged from 27 to 37, the mean number of oocytes retrieved per IVF cycle was 9, and the mean oocyte maturation rate was 70%. All MII oocytes inseminated by ICSI failed to fertilize. Sanger sequencing identified WEE2 mutations in all 4 women.

SAC proteins including Bub1B, Bub3, AURKA [25, 80, 82, 85–87], securin [82], and separase [88] are expressed in human oocytes. Cohesin is a multi-subunit protein complex made of four subunits. Four cohesin subunits are expressed in fetal [89] and adult human oocytes [90]: SMC1β, SMC3, REC8, STAG3. Tsutsumi et al. found an age-related decrease of REC8 and SMC1β protein in prophase I–arrested oocytes in women. This suggests that a decrease in oocyte cohesin levels may cause human oocyte aneuploidy [91, 92]. STAG3 mutations may cause POI [310].

## Oocyte Quality and IVM

One of the main clinical challenges facing assisted reproductive technology (ART) practitioners is the ability to select developmentally competent eggs and viable embryos [311]. The major problem is the unknown nature of oocyte competence also referred to as oocyte quality. Oocyte quality is defined as the ability of the oocyte to achieve meiotic and cytoplasmic maturation, fertilize, cleave, form a blastocyst, implant, and develop an embryo to term [312]. A major task for oocyte biologists is to find the oocyte mechanisms that control oocyte competence. Oocyte competence is acquired before and after the LH surge (Fig. 1). The development of oocyte competence requires successful completion of nuclear and cytoplasmic maturation [21]. Nuclear maturation is defined by cell cycle progression and is easily identified by microscopic visualization of the metaphase II oocyte. The definition of cytoplasmic maturation is not clear [5]. What are the oocyte nuclear and cytoplasmic cellular processes responsible for the acquisition of oocyte competence? What are the oocyte genes and how many control oocyte competence? Does LH signaling regulate oocyte competence? Can oocyte competence be improved?

Developmentally competent oocytes are able to support subsequent embryo development (Fig. 1). Oocytes progressively acquire competence during oogenesis. Several key oocyte nuclear and cytoplasmic processes regulate oocyte competence. The primary factor responsible for oocyte competence is probably oocyte ploidy and an intact oocyte genome. A mature oocyte must successfully complete two cellular divisions to become a mature healthy oocyte. During these cellular divisions, a high percentage of human oocyte chromosomes segregate abnormally resulting in chromosome aneuploidy. Oocyte aneuploidy is probably the major cause of reduced oocyte quality. Human oocytes are prone to aneuploidy. Over 25% of human oocytes are aneuploid compared with rodents 1/200, flies 1/2000, and worms 1/100,000. Many human blastocysts are aneuploid [313]. The major cause of human oocyte aneuploidy is chromosome non-disjunction [309, 314–317].

Approximately 40% of euploid embryos are not viable. This suggests that factors other than oocyte ploidy regulate oocyte competence. Other key oocyte nuclear processes include oocyte cell cycle mechanisms, oocyte spindle formation [305, 318], oocyte epigenetic mechanisms [319], oocyte DNA repair mechanisms, and oocyte meiotic maturation [12, 312]. Oocyte cytoplasmic processes include oocyte cytoplasmic maturation [5, 320], bidirectional communication between the oocyte and cumulus cells [101, 221, 321], oocyte mitochondria, oocyte maternal mRNA translation [322, 323], and oocyte biomechanical properties [81]. During the last 10 years, human oocyte gene expression studies have identified genes that regulate oocyte competence.

Microarray studies of human oocytes suggest that over 10,000 genes are expressed in MII oocytes [324, 325]. In an early microarray study, Bermudez et al. found 1361 genes expressed per oocyte in five MII-discarded oocytes that failed to fertilize [326]. These genes are involved in many oocyte cellular processes: cell cycle, cytoskeleton, secretory, kinases, membrane receptors, ion channels, mitochondria, structural nuclear proteins, phosphatases, protein synthesis, signaling pathways, DNA chromatin, RNA transcription, and apoptosis. Kocabas et al. found over 12,000 genes expressed in surplus human MII oocytes retrieved during IVF from three women [327].

Jones et al. studied human in vivo matured GV, MI, and MII oocytes and in vitro matured MII oocytes collected from patients undergoing ovarian stimulation and IVF [328]. Oocyte RNA was extracted and loaded on genome microarrays. They found an increase in gene expression in immature oocytes (GV, 10,962; MI, 12,329) and in vitro mature MII oocytes (9479) compared with in vivo matured MII oocytes (7546). In addition, 2000 transcripts were expressed 2-fold higher in IVM MII oocytes compared with in vivo matured MII oocytes. These genes are involved in oocyte cellular processes including DNA transcription, cell cycle control, cellular protein metabolism, and signal transduction. The authors suggest that increased gene expression in immature GV and IVM oocytes is due to a dysregulation of oocyte gene transcription which may reduce oocyte competence.

Zeng et al. studied the molecular basis of oocyte competence by comparing gene expression profiles of low competent, moderate competent, and high competent oocytes. They compared the gene expression profiles of oocytes from non-stimulated rhesus female monkeys (0.5–2-mm follicles; low competence), in vitro matured oocytes from females stimulated with FSH (moderate competence), and in vivo matured oocytes from female monkeys stimulated with FSH and HCG (3–7-mm follicles; high competence) and compared subsequent embryo development in these groups [329]. mRNA expression levels of 23 genes expressed in oocytes and embryos were analyzed with RT-PCR. The major finding was an increase in oocyte mRNAs in non-stimulated oocytes. The authors suggest a failure in normal transcriptional silencing in the oocytes from small < 2.0-mm follicles [330]. The embryos that developed from these low competent oocytes showed abnormally reduced gene expression. The authors conclude that altered transcription in non-stimulated oocytes from small follicles disrupts oocyte gene regulation, epigenetic modification, and metabolism. These oocyte abnormalities result in embryo developmental failure.

Grondahl et al. studied mRNA from normal donated MII oocytes from younger (< 36 years) and older [113–115] women using whole-genome sequence microarray [88]. A total of 7470 genes/single MII oocyte were identified. They identified several major functional gene categories including cell cycle, meiosis and mitosis, spindle function, anaphase-promoting complex (APC), electron transport chain-mitochondria, and oxidative stress. A total of 342 genes were differentially expressed and greater than 2-fold expression was found in 103 genes. These genes included the following: cell cycle (SMAD2, MAPK4, CDKNIC), meiosis (separase, EMEI), spindle function (MAD2LI, DOCR1), anaphase-promoting complex (ubiquilin I, ANAPC4, UBE4B, USP2, USP34, USP42, USP9X), mitochondrial (mitochondrial fission regulator 1—MtfrI), and DNA repair (nuclear autoantigenic sperm protein—NASP). These genes may contribute to the age-associated reduction in oocyte competence.

Grondahl et al. compared molecular pathways involved in oogenesis in oocyte from human primordial follicles and MII oocytes [82]. A total of 10,419 total genes and 2228 differentially expressed genes were identified in MII oocytes. The primary molecular pathways represented in MII oocytes showing 10-fold higher expression included cell cycle, oocyte maturation, and spindle organization, e.g., securin, cyclin B1, separase, CDC20, aurora kinase (AURKC), BMP15, GDF9, EGF, and EGFR. The authors state that accumulation of these specific transcripts in MII oocytes during oogenesis suggests that these genes may be important for MII oocytes to function. These genes may be required for the development of oocyte competence.

Riris et al. studied single human MII and GV oocyte mRNA levels of genes known to be functionally important contributors to oocyte quality in mice [80]. MII oocytes that failed to fertilize were studied. Ten genes were identified: CDK1, WEE2, AURKA, AURKC, MAP2k1, BUB1, BUB1B, CHEK1, MOS, FYN. mRNA levels were overall higher in GV oocytes than the MII oocytes. Individual MII oocyte mRNA abundance levels varied between patients. And gene expression levels widely varied among individual cell cycle genes in single oocytes. WEE2 was the highest expressed gene of this group. BUB1 expression was the lowest, approximately 100-fold lower than WEE2. Age-related changes were also observed. AURKA, BUB1B, and CHEK1 were lower in oocytes from an older patient than oocytes from a younger patient. The expression and abundance of these transcripts may reflect the level of oocyte competence.

Yanez et al. studied the mechanical properties, gene expression profiles, and blastocyst rate of 22 zygotes [81]. Mechanical properties at the zygote stage predicted blastocyst formation with 90% precision. Embryos that became blastocyst were defined as viable embryos. Single-cell RNA sequencing was performed at the zygote stage on viable and non-viable embryos. They found expression of 12,342 genes, of which 1879 were differentially expressed between both groups. Gene ontology clustering on the differentially expressed genes identified 19 functional clusters involved in oocyte cytoplasmic and nuclear maturation. At the zygote stage, all mRNAs, proteins, and cytoplasmic contents originate from the oocyte. The first two embryo divisions are controlled by maternal genes [331]. Gene deficiencies in cell cycle, spindle assembly checkpoint, anaphase-promoting complex, and DNA repair genes were identified in non-viable zygotes. Non-viable embryos had reduced mRNA expression levels of CDK1, CDC25B, cyclins, BUB1, BUB1B, BUB3, MAD2L1, securin, ANAPCI, ANAPC4, ANAPC11, cohesion complex genes including SMC2, SMC3 and SMC4, BRCA1, TERF1, ERCC1, XRCC6, XAB2, RPA1, and MRE11A. The authors suggest that reduced cell cycle transcript levels may explain abnormal cell division in cleavage embryos and blastocyst, and embryo aneuploidy.

Reyes et al. studied molecular responses in 10 oocytes (5 GV, 5 MII) from young women and 10 oocytes (5 GV, 5 MII) from older women using RNA-Seq sequencing (HiSeq 2500; Illumina) [79]. Patients were stimulated with FSH and triggered with HCG. GV oocytes were collected and used in this study. Some GV oocytes were placed in IVM media supplemented with FSH, EGF, and BMP. MII oocyte and GV oocyte total RNA was extracted, cDNA was synthesized and amplified and sequenced by single-cell RNA-Seq. Expressed genes were analyzed using weighted gene correlation network analysis (WGCNA). This identifies clusters of correlated genes. They found 12,770 genes expressed per oocyte, transcript abundance was greater in GV than MII oocytes, 249 (2%) were specific to MII oocytes, and 255 genes were differentially expressed between young and old MII oocytes. The major age-specific differentially expressed gene functional categories identified were cell cycle (CDK1), cytoskeleton, and mitochondrial (COQ3).

These human oocyte studies suggest that oocyte cell cycle genes are key regulators of oocyte competence. Cell cycle genes may be expressed 10-fold higher in MII oocytes compared with immature oocytes. These include securin, cyclin B1, separase, CDC20, aurora kinase (AURKC), BMP15, GDF9, EGF, and EGFR. The accumulation of these specific transcripts in MII oocytes during oogenesis suggests that these cell cycle genes may be required for the development of oocyte competence. Cell cycle gene expression levels are variable between MII oocytes. Not all MII oocytes are competent. A unique cell cycle gene expression profile may indicate MII oocyte competence. Cell cycle gene expression levels are reduced in abnormal blastocyst. These human oocyte studies suggest that cell cycle genes (Table 1) are required for the acquisition of oocyte competence, and that MII oocytes with abnormal cell cycle gene expression profiles develop abnormal embryos. Understanding the molecular determinants of oocyte quality is clinically important. The dramatic reduction of oocyte quality associated with advancing maternal age is a major cause of infertility [332]. Currently, there is no effective treatment to improve reduced oocyte quality.

## LH Signaling: Experimental Animal IVM Studies

In vitro maturation (IVM) oocyte culture systems have improved animal and human oocyte and embryo quality [6, 101]. The rationale of this approach is to synchronize oocyte nuclear and cytoplasmic maturation prior to completion of the first meiotic division. Premature resumption of meiosis is prevented to allow completion of normal nuclear and cytoplasmic maturation when oocytes are removed from follicles at oocyte retrieval. This allows oocyte cell cycle proteins to accumulate in the nucleus resulting in nuclear maturation. This also allows normal oocyte growth and duplication of cytoplasmic contents, i.e., ribosomes, Golgi, and mitochondria, and nuclear contents in preparation for the completion of the first and second meiotic cellular divisions of the oocyte.

This is accomplished, experimentally, by maintaining high cAMP levels in the cumulus-oocyte complex (COC) with phosphodiesterase inhibitors (PDE-I). Phosphodiesterases (PDE) breakdown cAMP which activates the oocyte CDK1/cyclin B resulting in resumption of meiosis and completion of the first meiotic division. Thus, immature incompetent oocytes can grow and develop into competent oocytes by allowing synchronization of nuclear and cytoplasmic growth. IVM studies demonstrate that cAMP-modulated IVM oocyte maturation rates, fertilization rates, and embryo cleavage rates can be improved. The cattle industry routinely utilizes IVM to produce healthy embryos. A total of 400,000 healthy cattle embryos were produced in 2013. Four IVM systems have been developed: standard IVM, biphasic (moderate cAMP), moderate induced (moderate cAMP), and high induced (high cAMP) [6, 101, 333].

Standard IVM protocols culture immature COCs in standard IVM media without cAMP modulators. IVM media are supplemented with FSH, LH, or HCG. Immature oocytes rapidly undergo spontaneous oocyte meiotic maturation. [165, 334]. Biphasic IVM systems utilize a phosphodiesterase inhibitor (PDE-I) for 24 h. This maintains moderate follicle cAMP levels which prevents oocyte nuclear maturation. This 24-h phase is followed by a PDE-I free 2nd phase which allows oocyte maturation to occur. The inhibition of oocyte nuclear maturation by cAMP was first demonstrated in the 1970s in mice and frogs [167, 335]. This approach improves mouse [336], bovine [337], and porcine [338] oocyte competence and embryo quality relative to standard IVM.

Induced IVM (high cAMP) protocols induce high cAMP levels, with cAMP stimulators, in the COC similar to the cAMP spike seen in vivo after the LH surge. Aktas et al. induced high cAMP levels in bovine oocytes with invasive adenylate cyclase. Ninety percent of the treated oocytes were maintained in meiotic arrest [339]. Funahashi et al. exposed porcine oocytes to the cAMP analogue dbc AMP [340]. Even though the oocyte maturation rate was not improved, oocyte quality was improved. Blastocyst rates were higher in the treated group compared with the untreated group (21.5% vs. 9%). Li et al. used forskolin (activates adenylyl cyclase) and IBMX (PDE-I) to increase follicle cAMP levels. This increased glutathione oocyte levels, reduced hydrogen peroxide levels, and reduced bovine oocyte oxidative stress. This improved oocyte and embryo quality [341].

Other novel IVM systems have also improved oocyte and embryo quality. EGF and AREG improve animal oocyte developmental competence [342]. Ritter et al. studied small (< 4 mm)- and medium-sized (> 4 mm) follicles, which represent low and moderate oocyte competence, respectively [343]. Denuded oocytes were matured in vitro in standard IVM media or IVM media supplemented with EGF. Cumulus cell EGFR gene expression and protein was measured with quantitative RT-PCR and western blot. Medium-sized follicles showed full cumulus cell expansion in response to EGF, while small follicles failed to expand. CC expansion gene (HAS2, PTGS2, TNFA1P6) mRNA expression was significantly lower in small follicles compared with medium follicles treated with EGF. EGFR mRNA expression levels were similar in small- and medium-sized follicles. EGFR protein and EGFR phosphorylation was increased in moderate- compared with small-sized follicles. EGF increased EGFR protein and EGFR phosphorylation in moderate-sized follicles, while EGFR protein and phosphorylation levels were undetectable in small-sized follicles. ERK1/2 phosphorylation was higher in moderate-sized follicles compared with small follicles. To determine whether native OSFs can cause CC expansion in small follicles, small follicles were co-cultured with denuded oocytes from medium-sized follicles and treated with EGF. Small follicles demonstrated full CC expansion. Native OSFs are probably acting via SMAD 2/3 since a SMAD antagonist prevented CC expansion. GDF9 and BMP15 did not induce CC expansion in small-sized follicles. Inseminated oocytes from moderate-sized follicles developed more blastocysts compared with oocytes from small follicles (45% vs. 15%). Small follicles treated with OSFs and EGF developed more blastocysts compared with those treated with EGF only (34% vs. 15%). The authors concluded that EGF and OSFs interact to improve oocyte competence.

OSFs improve oocyte and embryo developmental competence. Hussein et al. treated bovine COCs with GDF9 or BMP15 during IVM maturation [344]. The blastocyst rate was improved compared with controls (55% vs. 40%). GDF9 improved mouse fetal survival (40% vs. 20%) [345]. BMP15 improved oocyte and embryo quality by stimulating CC and oocyte gap junction activity [346].

CNP improves animal oocyte quality. Santiquet et al. preincubated murine COC with CNP, FSH, and BMP15 for 2 or 24 h [347]. Resumption of meiosis was prevented. Blastocyst rate (71.9% vs. 53.3%) and implantation rate (37.2% vs. 17.2%) were improved compared with controls after 96 h of culture.

These studies show that oocytes from larger follicles are more developmentally competent than oocytes from small follicles. The developmental competence of cultured oocytes can be improved with IVM protocols supplemented with cAMP modulators, EGF, AREG, OSFs, and CNP. The acquisition of oocyte competence is dependent on the accumulation of adequate cumulus cell EGFR, ERK1/2, and SMAD2/3 transcript levels and gap junction activity.

## LH Signaling: Experimental Human IVM Studies

Experimental human IVM studies performed during the last 10 years demonstrate that human oocyte and embryo quality can be improved (Table 2). Nogueira et al. performed the first IVM prematuration culture (PMC) human oocyte study. They studied human GV oocytes retrieved from 12-mm follicles or less after standard controlled ovarian hyperstimulation (COH) with FSH and triggered with HCG [93]. COCs were incubated with a PDE3-I for 24- or 48-h prematuration culture (PMC) period then washed and cultured in IVM media with FSH and EGF for 48 h. This was followed by insemination with ICSI; the embryos were grown for 3 days. In the control IVM group, COCs were grown in IVM media with FSH and EGF for 48 h. PDE3-I delayed meiotic progression, as 98% of the PDE3-I-treated GV oocytes remained arrested. PDE3-I-treated GV oocytes achieved higher maturation rates compared with control oocytes (67 vs. 46%; *p* = 0.01). The PMC treatment period did not improve fertilization or cleavage rates. In addition, higher oocyte maturation rates were found in COCs with moderate cell expansion compared with compacted COCs.

Shu et al. collected COCs from unstimulated and non-HCG-triggered 4–10-mm antral follicles by laparoscopy from 292 women mean age 34 [94]. A total of 730 COCs were cultured in IVM control media, or cilostamide (PDE3-I) alone, or forskolin (adenylate cyclase activator) alone, and combined cilostamide and forskolin in a 48-h PMC period followed by IVM for 24 h. Metaphase II oocytes were inseminated with ICSI and embryos were grown for 5 days. PDE3-I delayed meiotic progression. Oocyte maturation and embryo cleavage rates were similar in all groups (Table 2). The fertilization rate was increased in the combined groups compared with controls (52 vs. 76%). Gap junction communication (GJC) was prolonged 2-fold in the cilostamide + forskolin group compared with control. The authors concluded that the combined treatment, cilostamide and forskolin, increased follicle cAMP, delayed resumption of meiosis, and increased and maintaining GJC. This resulted in improved oocyte cytoplasmic maturation, and embryo quality as reflected in the increase in blastocyst rate. Further IVM studies are required to determine the optimal agents and dose and time intervals of PDE-I and AC activators.

Vanhoutte et al. stimulated patients with FSH 150 IU/day or Menopur (equal amounts of FSH- and HCG-driven LH activity) and triggered with HCG 5000 IU when two follicles reached a diameter of 20 mm [95]. GV and MII oocytes were retrieved from < 10-mm-diameter follicles for the study. Retrieved MII oocytes were the in vivo controls. IVM media, Tissue Culture Medium 199, was supplemented with EGF. PMC media were composed of basal medium (Tissue Culture Medium 199) supplemented with 0.8% human serum albumin plus PDE3-I (cilostamide). Cumulus-enclosed oocytes (CEOs) were embedded in an extracellular matrix (ECM) solution composed of collagen with PMC media for 24 h. The ECM solution allows the CEOs to maintain their three-dimensional (3D) tight structure. This preserves somatic cell-oocyte bidirectional communication which promotes oocyte quality. After the 24-h PMC period, COCs were cultured in IVM media for 24, 30, and 48 h. CEO controls were grown in IVM media only, for 48 h. MII oocytes were fertilized by ICSI. Fertilization was assessed at 16–18 h post-ICSI. Embryos were graded on days 2 and 3. PDE3-I delayed meiotic progression. Ninety percent of the oocytes grown in 3D PMC media were arrested at the GV stage. The MII rate (60.6% vs. 81.6%; *p* < 0.05), fertilization rate (27.3% vs. 59.6%), and embryo cleavage rate (27.3% vs. 55.6%) were all higher in the 3D PMC group compared with the IVM conventional group (Table 2). Electron microscopy studies found that the 3D PMC method maintained the CEO 3D structure, cumulus-oocyte contacts were maintained, and transzonal projections were identified. Lucifer yellow dye coupling assay identified more functional gap junctions in the 3D PMC group compared with controls. They concluded that the 3D PMC period improved oocyte maturation and embryo cleavage rates reflecting an improvement in oocyte quality, and oocyte meiotic and cytoplasmic maturation. This improved oocyte quality may be related to preservation of cumulus-oocyte gap junctions.

Novel IVM culture systems supplemented with growth factors and other signaling molecules improve oocyte quality. Goud et al. studied the effect of EGF on oocyte quality. They obtained 289 spare germinal vesicle (GV) oocytes from 92 infertile patients stimulated with human menopausal gonadotropin (HMG), which were retrieved 36 h post-HCG 10,000 IU, and then inseminated with ICSI [97]. The mean age of the patients was 31.8 years. GV oocytes were cultured in IVM media supplemented with EGF for 12, 24, and 30 h. GV oocytes were divided into two groups: group I, cumulus-denuded oocytes with and without EGF and group II, intact oocytes with and without EGF. At 30 h, the MII rate in the denuded was higher in the oocytes supplemented with EGF (64.3% vs. 33.9%) (Table 2). The fertilization rate in the denuded group was similar to that in the EGF- and non-EGF-supplemented oocytes. In the intact group, the fertilization rate was higher in the EGF-supplemented oocytes (71.7% vs. 45.6%). The day 3 cleavage rate was overall higher in the oocyte-intact EGF group. They concluded that retaining cumulus cells and adding EGF improve the MII, fertilization, and cleavage rates. This suggests improved oocyte meiotic and cytoplasmic maturation, and overall improved oocyte quality.

Ben-Ami et al. reported improved MII and embryo cleavage rates with IVM media supplemented with EGF and AREG [98]. Thirty patients were treated with standard COH stimulation, retrieval, and ICSI. A total of 105 GV oocytes were cultured with IVM media supplemented with both AREG and EREG for 24 h. The MII rate was increased in the supplemented group (75.5 vs. 36.5%, *p* < 0.001). The fertilization and cleavage rates were not improved with supplementation; however, the higher MII rate allowed more embryos to be produced. This was the first study to show a positive effect of EGF and AREG on human oocyte maturation rate.

Sanchez et al. reported a new IVM strategy for immature oocytes retrieved from small follicles (2–6 mm) [99]. Thirty patients with PCOS, average age 28.9, were stimulated with Menopur for 3 days. HCG trigger was not given. All follicles were < 10 mm prior to retrieval. Control COCs were cultured in IVM media supplemented with HMG and HCG, and cultured for 30 h. In the PMC/CNP group, COCs were collected in media supplemented with PDE3-I (IBMX), then washed and cultured in IVM media supplemented with FSH and CNP for 24 h, and then washed and cultured in IVM media supplemented with AREG and FSH for 30 h. MII oocytes were inseminated using ICSI. All experimental blastocysts were biopsied and analyzed for aneuploidy by next-generation sequencing (NGS). The oocyte maturation rate (48% vs. 70%), the day 3 good-quality embryo rate (23% vs. 43%), and the blastocyst rate (8% vs. 18%) were all increased in the PMC group compared with the control IVM group (Table 2). Transzonal projections (TZPs) were maintained in the PMC group which may explain improved oocyte competence in this group. The blastocyst aneuploidy rate (3 aneuploid/10) was not increased compared with standard ART. IVM/PMC with CNP improved oocyte maturation, fertilization, and blastocyst rates. This suggests that oocyte meiotic and cytoplasmic maturation and oocyte quality are improved. Whether IVM/PMC systems can reduce embryo aneuploidy rates is not known.

Madkour et al. reported a new rescue IVM approach [100]. All patients were stimulated with standard FSH doses, triggered with HCG, and oocyte retrieval was performed 36 h post-HCG. MII oocytes were inseminated with ICSI and cultured 5 or 6 days. They randomized 150 GV oocytes from 47 patients with PCOS. Immature GV oocytes were randomized to four groups. The simple-IVM (S-IVM) protocol contained standard IVM media. In the autologous follicular fluid (AFF-IVM) protocol, AFF was added to standard IVM media. AFF was collected from the study patients during the oocyte retrieval. In the heterologous follicular fluid (HFF-IVM) protocol, HFF was added to standard IVM media. HFF was taken from 7 women without PCOS with subsequent 100% MII maturation. HFF was added to standard IVM media. In the heterologous follicular fluid/CGC (HFF/CGC-IVM) protocol, HFF and cumulus-granulosa cell (CGC) supernatant were added. CGC supernatant was obtained from 7 women without PCOS. FF was collected at egg retrieval, centrifuged, CGCs were collected and cultured for 3 days, and then supernatant was collected and added to IVM media. Immature oocytes were cultured for 24 h. All IVM-matured MII oocytes were inseminated with ICSI and cultured to day 5 or 6. The MII, cleavage, and blastocyst rates were higher in the HFF/CGC-IVM group compared with standard IVM (Table 2). The CGC supernatant contains growth factors and cytokines which may be responsible for the improvement in maturation rate and embryo quality. The MII maturation rate in the HFF/CGC group of 79% and 65% day 5 blastocyst rate are similar to standard IVF MII and blastocyst rates.

Spits et al. treated 16 young (mean age was 28.7) PCOS women with Menopur 150 IU/day starting on day 3 of the cycle for 3 days; ultrasound was performed on day 6, and retrieval for immature oocytes was performed on day 7 on all patients 42 h after the last dose of Menopur [96]. HCG trigger was not given. COC oocytes were treated with PDE3-I (IBMX) for 1 h in standard IVM media. COCs were then washed and placed in IVM culture media supplemented with FSH for 40 h, then the cumulus layer was removed, oocyte maturation was assessed, and ICSI was performed. The oocyte maturation rate was 50.2% (120/239), the fertilization rate was 68.3% (82/120), and the day 3 cleavage good-quality embryo rate was 30.5% (25/82). Chromosome analysis was performed on single blastomeres. Eighteen embryos were dissociated, individual blastomeres were washed, 136 cells were successfully amplified and analyzed with aCGH, and 123 cells gave results. Sixty-one percent (11/18) of the IVM embryos were mosaics. This is similar to the day 3 embryo mosaic rate in standard IVF.

These human IVM studies demonstrate that human oocyte quality can be improved. IVM/PMC culture systems supplemented with PDE-I, EGF, AREG, or CNP improve oocyte maturation, cleavage rate, and blastocyst rate compared with standard IVM systems (Table 2). IVM/PMC with PDE-I delays resumption of meiotic maturation by increasing cAMP in the COC. This allows the cytoplasmic and nuclear contents of the oocyte to grow and develop, thus allowing time for oocyte meiotic and cytoplasmic maturation. Improved oocyte meiotic maturation resulted in MII rates approaching 80%. As a result of improved oocyte cytoplasmic maturation, the IVM fertilization rate approached 80%, and cleavage rate approached 50–70%. These IVM rates suggest that IVM may become a routine alternative ART for some patients in the near future. Oocyte and follicle gap junction activity and transzonal projections allowing bidirectional communication between oocyte-granulosa cells may be important mechanisms underlying oocyte quality. These studies also suggest that IVM/PMC system embryo aneuploidy rates and mosaicism are not increased compared with standard IVF.

### Clinical Human IVM

The first human oocyte IVM studies were performed over 50 years ago [334, 348]. The first successful human IVM births were reported over 25 years ago [349, 350]. During the first 20 years of IVM, the pregnancy rates from IVM cycles were reduced by half compared with standard IVF [351–355]. The reduced IVM pregnancy rate was attributed to asynchrony between nuclear and cytoplasmic maturation [356].

Recent IVM studies demonstrate improved outcomes [357–360]. Walls et al. recently performed the first study that compared IVM and standard IVF blastocyst development [361]. They studied 56 PCOS patients (80 cycles) who were treated with IVM and 65 PCOS patients (98 cycles) treated with standard IVF. The IVM patients were treated with Gonal-F (recombinant FSH) 150 IU/day started on cycle day 2 after transvaginal ultrasound and was continued for 3–6 days. Transvaginal ultrasound was repeated on day 6 of the cycle, and oocyte retrieval was performed within 72 h after a 10-mm follicle was observed. COCs were cultured for 24 h in G-2Plus media which is a bicarbonate-buffered media with hyaluronan and maternal serum. This was supplemented with FSH and hCG. MII oocytes were inseminated with ICSI. The total number of oocytes retrieved per patient was similar in the IVM and IVF groups (13.2 vs. 16.6%). The maturation rate (73 vs. 80%) and fertilization rate (68 vs. 77%) were superior in the IVF group, and the good-quality blastocyst rate was the same in both groups (38 vs. 40%). The live birth rate per transfer was superior in the IVF fresh transfer group compared with the IVM group (18.8 vs. 31.0%), and similar in the frozen IVM and IVF groups (33.9 vs. 29.9%). The cumulative live birth rate per egg retrieved was higher in the IVF group (41.3 vs. 55.1%). The miscarriage rate was higher in the fresh transfer IVM group compared with the fresh IVF group (36.8 vs. 19.0%), and lower in the frozen transfer IVM group compared with the frozen IVF group (4.5 vs. 18.6%). The birth weights and preterm labor rate were similar in both groups. The authors concluded that the efficiency gap between IVM and IVF is closing, and that IVM should be recommended for PCOS patients who have experienced OHSS with standard IVF.

Birth outcomes are similar in IVM and IVF children. Preterm birth rates and newborn birth weights are similar in IVM and IVF births [362, 363]. Congenital birth defects are not increased in IVM children [364, 365]. Childhood development is similar in IVM and IVF. Roesner et al. studied 21 children conceived by IVM [366]. At birth, weight, length, and head circumference were similar in IVM children compared with IVF controls. At age 2, weight, length, and head circumference and cognitive development were similar in IVM compared with IVF controls.

## Summary

Here, we reviewed human LH signaling oocyte meiotic maturation studies. We found 89 human studies in the literature on this topic. These studies identified and characterized 24 LH signaling proteins involved in oocyte meiotic maturation (Table 1). Coticchio et al. recently reviewed human oocyte maturation and similarly found < 50 human studies in the literature on this topic [5]. These human studies suggest that the primary targets of the LH signal in the follicle are the CNP/NPR2 system, the EGF/EGF receptor network, and gap junctions. The primary target of the LH signal in the oocyte is the MPF (CDK1/Cyclin B1). The activated MPF initiates resumption of meiosis by phosphorylating downstream proteins including SAC proteins, APC proteins, separase, securin, and cohesin. How these downstream proteins induce resumption of meiosis and completion of the first meiotic division including germinal vesical breakdown, chromosome condensation, and extrusion of the first polar body in humans is not known. Additionally, these LH signaling molecules may predict oocyte quality, a critical issue in assisted reproductive technology (ART); however, a reliable marker of oocyte quality still has not been identified.

These LH signaling pathway molecules also regulate oocyte competence. Human oocyte gene expression studies suggest that oocyte cell cycle proteins targeted by the LH signal are key regulators of oocyte developmental competence. Differences in cell cycle gene expression have been identified between human immature oocytes from primordial follicles and MII oocytes. Grondahl et al. found differences in securin, cyclin B1, separase, CDC20, aurora kinase (AURKC), BMP15, GDF9, EGF, and EGFR [82]. Riris et al. studied single human MII and GV oocyte cell cycle mRNA levels and found differences in CDK1, WEE2, AURKA, AURKC, MAP2k1, BUB1, BUB1B, CHEK1, MOS, and FYN [80]. Yanez et al. found differences in cell cycle gene expression profiles of viable and non-viable zygotes including CDK1, CDC25B, cyclins, BUB1, BUB1B, BUB3, MAD2L1, securin, ANAPCI, ANAPC4, ANAPC11, cohesion complex genes including SMC2, SMC3, and SMC4, BRCA1, TERF1, ERCC1, XRCC6, XAB2, RPA1, and MRE11A [81]. Reyes et al. studied cell cycle expression profiles in 10 oocytes (5 GV, 5 MII) from young women and 10 oocytes (5 GV, 5 MII) from older women [79]. They found differences in CDK1. These studies suggest that the expression and abundance of these oocyte cell cycle transcripts may determine whether an oocyte acquires competence, and whether it is able to form a viable embryo.

Human oocyte quality can be improved with IVM/PMC manipulation of the LH signaling pathway (Table 2). Human oocyte IVM cultures supplemented with PDE-I [95], AREG [98], CNP [99], and heterologous follicular fluid/CGC [100] improve oocyte maturation and embryo quality. Experimental IVM oocyte maturation rates (range, 70–81.6%) are approaching standard IVF maturation rates. Clinical IVM neonatal outcomes are similar to standard IVF outcomes. The IVM aneuploidy rate is not increased in day 3 embryos [96] and blastocysts [99]. These experimental IVM protocols may soon be introduced into clinical IVM practice further improving clinical IVM.

## Conclusion

We found 89 papers in the literature that studied human LH signaling oocyte meiotic maturation. These studies identified 24 proteins involved in this process (Table 1). The proteins expressed in the human ovarian follicle compartment are signaling proteins, while the proteins expressed in human oocytes are primarily cell cycle proteins. The primary targets of the LH signal in the follicle are the CNP/NPR2 system, EGF network, and gap junctions (Fig. 2). The primary target of the LH signal in the oocyte is the CDK1/Cyclin B complex. These follicle/oocyte proteins are vitally important. They regulate human oocyte meiotic maturation, oocyte quality, and embryo quality. Remarkably, human oocyte and embryo quality is improved using IVM/PMC cumulus-oocyte culture systems that manipulate the LH signaling pathway (Table 2). The studies reviewed were mostly published during the last 10 years. Human oocyte maturation studies are very limited in number. Human oocyte research is only beginning. Hopefully, human oocyte and embryo research will continue to increase in the future so that further insight into the cellular mechanisms that regulate oocyte and embryo quality can be acquired.

## Abbreviations

APC/C: anaphase-promoting complex or cyclosome
AREG: amphiregulin
BMP: bone morphogenetic protein
Bub: budding uninhibited by benzimidizole protein
CC: cumulus cells
CDC: cell division cycle
CDK1: cyclin-dependent kinase 1
CNP: C type natriuretic peptide
CGC: cumulus-granulosa cell
COC: cumulus-oocyte complex
EGF: epidermal growth factor
EGF-LP: epidermal growth factor-like peptides
ERK: extracellular signal–regulated kinases
FSH: follicle-stimulating hormone
GC: granulosa cells
GDF9: growth differentiation factor 9
GJ: gap junctions
GPR3: G protein–coupled receptor 3
GVBD: germinal vesicle breakdown
Has2: hyaluronan synthase 2
HMG: human menopausal gonadotropin
IVM: in vitro maturation
LH: luteinizing hormone
MAD: mitotic arrest–deficient protein
MAPK: mitogen-activated protein kinases
MI: metaphase I
MII: metaphase II
NPPC: natriuretic peptide precursor C
NPR2: natriuretic peptide receptor 2
OQ: oocyte quality
OSF: oocyte-secreted factor
PDE: phosphodiesterase
PP: protein phosphatase
Ptgs2: prostaglandin-endoperoxide synthase 2
SAC: spindle assembly checkpoint
TGF: transforming growth factors
TKR: tyrosine kinase receptor
Tnfaip: tumor necrosis factor alpha–induced protein 6
TM: transmembrane
TZP: transzonal projections
